# Viruses of Polar Aquatic Environments

**DOI:** 10.3390/v11020189

**Published:** 2019-02-22

**Authors:** Sheree Yau, Mansha Seth-Pasricha

**Affiliations:** 1 Integrative Marine Biology Laboratory (BIOM), CNRS, UMR7232, Sorbonne Université, 66650 Banyuls-sur-Mer, France; sheeyau@gmail.com; 2 Institute of Earth, Ocean, and Atmospheric Sciences, Rutgers University, New Brunswick, NJ 08901, USA; 3 Department of Ecology, Evolution, and Natural Resources, Rutgers University, New Brunswick, NJ 08901, USA

**Keywords:** arctic, antarctica, viruses, freshwater, saline, DNA viruses, RNA viruses, polar regions

## Abstract

The poles constitute 14% of the Earth’s biosphere: The aquatic Arctic surrounded by land in the north, and the frozen Antarctic continent surrounded by the Southern Ocean. In spite of an extremely cold climate in addition to varied topographies, the polar aquatic regions are teeming with microbial life. Even in sub-glacial regions, cellular life has adapted to these extreme environments where perhaps there are traces of early microbes on Earth. As grazing by macrofauna is limited in most of these polar regions, viruses are being recognized for their role as important agents of mortality, thereby influencing the biogeochemical cycling of nutrients that, in turn, impact community dynamics at seasonal and spatial scales. Here, we review the viral diversity in aquatic polar regions that has been discovered in the last decade, most of which has been revealed by advances in genomics-enabled technologies, and we reflect on the vast extent of the still-to-be explored polar microbial diversity and its “enigmatic virosphere”.

## 1. Introduction

Microorganisms are at the heart of the Arctic and Antarctic food webs. These polar environments contain a diverse range of bacterial, archaeal, and eukaryotic microbial communities that, along with viruses, comprise important components of the polar ecosystems [1,2,3]. They are found in a range of habitats, including subglacial lakes and cryoconite holes, making the cold biomes of these polar regions replete with metabolically diverse microorganisms and sites of active biogeochemical cycling [4,5,6]. These environments, that cover approximately one-fifth of the surface of the Earth and that are inhospitable to human life, are home to unique microbial communities [1,6,7]. The resident microbiota of the two regions has a similarity of only about 30%—not necessarily surprising given the limited connectivity of the polar oceans and the difference in freshwater supply, coming from glacial melts and rivers that drain into the Southern Ocean and the Arctic Ocean, respectively [7]. The separation is not just by distance: Antarctica is surrounded by the Southern Ocean that is driven by the strong Antarctic Circumpolar Current, whereas the Arctic is ringed by landmasses. Such different topographies resulted as the two continents moved to the opposite polar regions of the planet ~40–25 million years ago. Magnetic and gravity data point to the evolution of the Arctic, driven by the Amerasian and Eurasian basins, from 145–61 million years ago to a cold polar region of water and ice surrounded by land [8,9,10]. Antarctica was formed from the breakup of the super-continent, Gondwana, a landmass surrounded by the Southern Ocean [1,11]. The Antarctic continent is permanently covered with glacial ice, with only 0.4% of its area comprising exposed land dotted with lakes and ponds.

Microbes, both prokaryotic and eukaryotic that are present in these environments, are largely different between the two poles [7,12]. For example, 78% of bacterial operational taxonomic units (OTUs) of surface water communities of the Southern Ocean and 70% of the Arctic Ocean are unique to each pole [7]. Polar regions are variable in time and space—analysis of the V6 region of the small subunit (SSU) rRNA gene has resulted in ~400,708 gene sequences and 11,441 OTUs from 44 polar samples of the Arctic and the Southern Ocean. These OTUs cluster separately for the two polar regions and, additionally, exhibit significant differences in just the polar bacterioplankton communities from different environments (coastal and open ocean) and different seasons [7].

**Figure 1:**
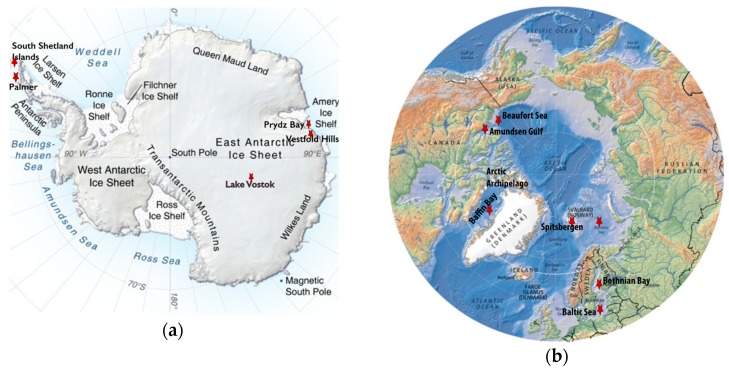
Maps indicating the locations of the polar regions described in this review (red stars) that have been sampled for viruses. When multiple different sites were sampled in a region, they are listed in parentheses as follows. (**a**) A topographic map of Antarctica modified with labels identifying the South Shetland Islands (Livingston Island: Limnopolar Lake and nine freshwater lakes on Byers Peninsula), the Vestfold Hills (Ace, Organic, and Deep Lakes), Palmer Station on the West Antarctica Peninsula, Prydz Bay, and Lake Vostok. (**b**) A map of the Arctic modified to include the Arctic Archipelago, Baffin Bay, Beaufort Sea, Amundsen Gulf, Svalbard Archipelago highlighting Spitsbergen Island (six freshwater lakes: Linnevatnet, Borgdammane, Tunsjoen, Tenndammen, IR2, and Nordammen; Kongsfjorden; and Storfjorden), Barents Sea, Bothnian Bay, and the Baltic Sea. Image Credits: The Antarctica map was created by Philippe Rekacewicz and Emmanuelle Bournay, UNEP/GRID-Arendal, available at grida.no. The Arctic map was created by Hugo Ahlenius, UNEP/GRID-Arendal, available at grida.no.

Viruses are the “most abundant biological entities on the planet” [13], particularly in the oceans [13,14], which make up ~70% of the Earth’s surface. The discovery that there are ~10^8^ viruses in every milliliter of water [15] three decades ago gave impetus to understand their diversity and role in the marine environment [14]. They are now considered to play key roles in marine ecosystems by controlling microbial community dynamics, host metabolic status, and biogeochemical cycling via lysis of hosts [13,14,16]. In the polar regions characterized by truncated food webs, the role of viruses in ecosystem function is likely even greater, yet their diversity is still relatively underexplored, and the way in which they affect polar communities is not well understood [5], particularly in nutrient cycling [3,17,18]. This review brings together literature from the last decade on the polar viral diversity associated with aquatic microbial communities, focusing on metagenomic and molecular fingerprinting studies. Although relatively few polar viral diversity studies have been conducted (see the maps in Figure 1 for polar virus sampling sites), they have provided an unprecedented level of insight into the virus world and helped to circumvent the difficulty of viral isolation in these hard-to-access environments. While it is still an outstanding question if polar viral species differ significantly from low latitude species, or if they differ between the poles, what stands out is that the assembly of large viral genomic fragments from polar metagenomes has been highly successful. This is despite the smaller sampling effort compared to temperate marine metagenomes and perhaps reflects the heightened importance of viruses as primary predators, especially in polar lakes that lack metazoan grazers. To understand viral diversity in the polar areas, this review breaks the available knowledge by polar regions and genetic material of viruses (DNA and RNA).

## 2. Antarctica

Microbial diversity in Antarctica follows seasonal variations influenced by temperature, light, wind, nutrients, and salinity depending on the site of sampling [4,19]. The waters surrounding the West Antarctica Peninsula (WAP) are dominated by diatoms in the austral summer, whereas other microbial eukaryotes, like cryptophytes and haptophytes, can form major blooms from the austral spring to fall [5,20,21,22,23,24]. Along coastal regions of the WAP, bacteria and archaea also show differential abundances in the summer and winter months. Their diverse metabolisms, which include chemoheterotrophy, photoheterotrophy, photosynthesis, and chemolithoautotrophy, vary based on seasonal light and temperature changes [3,19]. The lakes of Antarctica range from freshwater to hypersaline, and ice-free to permanently ice-covered. These environments of low light, low temperature, and mostly low inorganic nutrients are characterized by their truncated food webs lacking in metazoan life and harbor instead protozoa, algae, bacteria, archaea, fungi, and viruses [3,18,25]. In particular, archaea are found to dominate in the hypersaline lakes [26].

Viruses of Antarctic aquatic environments are not only diverse and abundant [27], but also have been shown to shift in lifestyle, similar to their microbial hosts, from pseudolysogenic to lytic when climate conditions favor microbial growth [5,18,21]. Metagenomic studies have revealed a high viral diversity, novel viruses and virophages, and indicated host–virus interactions that may regulate light-driven microbial loop dynamics [17,21,28,29,30,31,32,33,34,35]. This diversity appears unique to Antarctica, as evidenced by the *Tara* Oceans Virome dataset (TOV) where analyses of protein clusters from contigs up to 100 kb indicated that viral populations in Antarctica diverged from the rest of the world (26 total including surface and deep ocean sites) [36]. Figure 1a shows sampling sites in Antarctica mentioned in this review.

### 2.1. DNA Viruses in Antarctica

An unexpectedly rich genetic diversity of single-stranded DNA (ssDNA) and double-stranded DNA (dsDNA) viruses has been reported in the freshwater Limnopolar Lake [17,29,31] located on Livingston Island, in Byers Peninsula (South Shetland Islands), West Antarctica. Viral abundances were found to vary seasonally with the lake, which is ice-covered in spring and ice-free in summer. Despite the low nutrients, dim light, and the approximately nine months of ice-cover per year followed by a short ice melt and an ensuing high ultraviolet radiation when exposed in summer [37], the lake harbors diverse populations of bacteria, algae, protozoa, and rotifers [37]. Electron microscopy, flow cytometry, and pyrosequencing of viral DNA revealed viral morphologies and viral genotypes from 12 virus families [17], the first study to identify such high viral diversity in high latitude environments. Typically, high latitude ecosystems have been reported to have low diversity [38]. However, a phenomenally high species richness comprising 5130 viral genotypes in the spring and 9730 in the summer was reported in Limnopolar Lake. This was more than an order of magnitude higher than that reported for any other freshwater virome in lower latitudes (253–787 genotypes) and amongst the highest reported in seawater viromes; for example, the Sargasso Sea was estimated to have 5140 genotypes and the Gulf of Mexico 15,400 from assembled metagenomic reads [17,38]. However, only ~13% of these sequences showed similarity to NCBI Genbank sequences and ~28% to metagenomic open reading frames (ORFs).

A change in viral composition was evidenced by a shift in viral particle size that accompanied a change in season—viral particles of diameters <30 nm dominated in the spring, whereas >50-nm-sized particles including tailed phages and putative phycodnaviruses with a capsid size of ~150 nm dominated in summer [17]. Of the ssDNA spring viruses, other than ones related to the members of *Clamydiamicrovirus* genus, many were curiously related to members of the *Circoviridae*, *Geminiviridae*, *Nanoviridae*, and satellites. Such viruses are typically associated with multicellular eukaryotes such as mammals, birds, and plants, which are absent in this lake. The abundance of ssDNA sequences allowed assembly of circular ssDNA contigs with a similarity to nanoviruses and satellites, and circoviruses; this included two unidirectional ORFs with no similarity to previously described circular ssDNA viruses, as well as an additional 25 circular DNA contigs with no similarity to known genomic sequences [17]. Despite the possibility of preferential amplification of ssDNA as the viromes were generated using the Phi29 polymerase that is known to have this bias, this study identified the presence and abundance of unique viruses in Antarctica. In particular, it highlighted the dominance of viruses that infect eukaryotic microbial hosts and not prokaryotic hosts, unlike other aquatic viromes [17]. A secondary population of dsDNA bacteriophages of the order *Caudovirales* was also present in the spring viromes, but the summer virome was dominated by dsDNA viruses belonging to the *Phycodnaviridae*; viruses related to members of the *Caudovirales* and *Mimiviridae* were also identified. While a distinct Antarctica prasinovirus was identified [17], ~87% of the phycodnavirus sequence matched OtV5, a prasinovirus species that infects *Ostreococcus tauri* [39]. This Limnopolar Lake summer viral community was resequenced using Illumina technology and produced additional contigs: Ant-0, similar to the phycodnavirus, *Bathycoccus prasinos* RCC1105 virus BpV1; Ant-53, with regions similar to the mimivirus, *Cafeteria roenbergensis* virus BV-PW1 (CroV); and the phycodnavirus, *Phaeocystis globosa* virus (PgV) [31].

Diverse viruses, unlike any from previously sampled freshwater systems of higher latitudes, were identified in nine polar freshwater lake viromes along a latitudinal transect spanning ~650 km of Byers Peninsula, Livingston Island (North-West Antarctica Peninsula). These viruses shared 3–27% functional similarity when compared with reference viromes from other polar and other global freshwater environments [29] when analyzed using a coarse-grained dinucleotide odds ratio, cross-tblastx analysis (crAss), and cross assembly [29,31]. These analyses clustered viral communities based on the environments they were isolated from, clearly distinguishing the two polar communities. A large proportion of the Antarctic freshwater viruses are unique and not related to known viral families, pointing to the vastly unexplored virosphere. A large proportion of these reads from the lake viromes were found to be ssDNA viruses (unclassified ssDNA and *Circoviridae*-like) with a significant presence of viruses assigned to the order *Caudovirales* in some lake communities. *Phycodnaviridae*-related sequences and unclassified ssRNA viruses were detected in all viromes. However, a latitudinal gradient of viral diversity was not observed in these Antarctica Peninsula freshwater lakes [29].

A change in viral communities was also demonstrated by metagenomic analyses of microbial communities over a spring-to-summer transition in the coastal WAP that revealed the presence of temperate dsDNA phages infecting bacteria and archaea [21]. This work elegantly showcased a difference in the bacterial community composition upon induction of temperate viruses in the austral spring and summer using mitomycin C, which, in turn, led to a decrease in gammaproteobacterial abundance with a simultaneous increase in *Flavobacteria*. Viruses switched from lysogeny to lysis based on the physiological state of the microbial host as it transitioned from harsh winter conditions. Although only 5–7% of viruses could be taxonomically annotated, podoviruses dominated the temperate-enriched metagenomes and myovirus-like sequences abounded in the lytic-enriched metagenomes [21]. The work further proposed the role of temperate viruses in manipulating the fitness of infected hosts—thus contextualizing a critical role of viruses in the temporal uncoupling of bacterial production and primary production in the Southern Ocean [40].

Antarctica is also home to virophages; viruses that utilize giant viruses in the *Acanthamoeba polyphaga* mimivirus (APMV) family to replicate, thereby acting as a virus of a virus [33,34]. Metaproteogenomic analyses of Organic Lake were performed on biomass collected on 3-, 0.8-, and 0.1-μm filters, the latter of which consisted of an unusually high proportion of viral signatures [34]. Organic Lake is a 7-m deep hypersaline meromictic lake in the Vestfold Hills of East Antarctica where surface waters vary in temperature from −14 to +15 °C and all primary production seems to originate from only a few eukaryotic algal species. Large scaffolds assembled from the 0.1-µm size fraction were sorted on the basis of GC content and coverage. High-GC scaffolds contained phage homologs, and low-GC scaffolds contained phycodnavirus homologs; specifically, they were similar to viruses infecting eukaryotic algae (CeV, PgV and PoV) that are also related to APMV. The latter were near-complete genomes termed OLPVs (Organic Lake phycodnaviruses) making up 60% of the assembled reads, most likely infecting the prasinophyte alga *Pyramimonas* that was also detected in the lake [34]. A scaffold corresponded to a circular dsDNA virophage termed OLV (Organic Lake virophage) similar to the Sputnik virophage of APMV [41]. OLV was proposed to utilize the OLPV as its viral host and thus reduce predation of host algal cells and stimulate repeated algal blooms during the polar summer months.

Surface water metagenomes from Ace Lake, a meromictic lake with 2% salinity also located in the Vestfold Hills, similarly contained virophage signatures [33,34]. It is covered with ~2 m of ice for nearly the entire year, usually thawing in the austral summer month of January, and has a sharp chemocline corresponding to an O_2_–H_2_S interface. Phytoplankton comprising *Pyramimimonas gelicola*, *Mesodininium rubrum*, diatoms, and photosynthetic cyanobacteria dominate the oxic zone, contributing to primary production that peaks during the ~6-week summer period. Green sulfur bacteria of the genus *Chlorobium* form a dense layer at the O_2_–H_2_S interface [42]. Virus concentrations in Ace Lake’s mixolimnion are estimated to be 8.9 × 10^9^ L^−1^ to 61.3 × 10^9^ L^−1^ with virus-to-bacterium ratios (VBR) of 30.6–80.0 [43]. Hence viruses are likely key drivers of biogeochemical cycling in this environment [44]. Indeed, mining for virophages in the CAMERA (Cyberinfrastructure for Advanced Marine Microbial Ecology Research and Analysis) [45] metagenomic database retrieved a 17,767-bp complete genome of a dsDNA Mavirus-like virophage, termed ALM, for Ace Lake Mavirus. Like the Sputnik virophage [46], Mavirus is a parasite of the giant *Cafeteria roenbergensis* virus (CroV) [47]. Giant phycodnaviruses are in abundance in the oxygen-rich layer [33] and are potential host viruses for ALM. Phylogenetic analysis indicated that Mavirus and ALM shared 13 homologous genes, five of which were the conserved virophage genes: FtsK-HerA family DNA packaging ATPase (ATPase), DNA helicase/primase (HEL/PRIM), cysteine protease (PRSC), major capsid protein (MCP), and minor capsid protein (mCP). Additionally, they shared three putative genes: GIY-YIG endonuclease, rve super family integrase, and protein-primed B-family DNA polymerase [46]. A more recent and comprehensive search for virophage and protein sequences similar to Polintons (large eukaryotic transposons) in the CAMERA and NCBI Whole Genome Shotgun Contig Database identified a new and diverse group of putative viruses called Polinton-like viruses (PLVs), one of which originated from Ace Lake (named ACE) [48]. PLVs have a genome size of 15–30 kb that may or may not encode integration elements; however, as they possess genes similar to the MCP morphogenetic module, a packaging ATPase, and a maturation protease, they are proposed to be bona fide viruses [48].

Another lake situated in the Vestfold Hills, East Antarctica, is Deep Lake, which stays liquid at temperatures as low as −20 °C due to its high salinity [49]. Deep Lake is dominated by haloarchaea throughout its 36-m depth. Approximately 72% of the entire lake community comprises haloarchaeal species that are in order of decreasing abundance: 44% *Halohasta litchfieldiae* strain tADL, 18% strain DL31, 10% *Halorubrum lacusprofundi* strain ACAM34, and 0.3% *Halobacterium sp.* strain DL1 [26]. In contrast, the most abundant bacterial taxa are *Halomonas* spp., at only ~0.8% of the Deep Lake community [26,32]. The haloarchaea exhibit ecotype distinction at the genus and strain level yet share long (~35 kb) and conserved (up to 100%) high identity regions (HIRs) that appear to be mobilized by viral transduction and suggest their viruses have extremely broad host ranges. Eight different haloarchaeal dsDNA viruses resembling bacteriophages of the order *Caudovirales* were identified in Deep Lake metaproteomes from their MCPs [32]. Based on MCP alignments, five of these were identified as siphoviruses belonging to subgroups of the unclassified dsDNA haloviruses HCTV-1, HCTV-2, HHTV-1, and *Halorubrum* virus CGΦ46. Metagenomic assemblies also indicated the presence of putative lytic head-tailed viruses eHP-12 and eHP-6, and one virus that matched the myovirus VBM1 infecting *Vibrio parahemolyticus*, a marine gammaproteobacterium. One of the most interesting discoveries of this study was the mosaic nature of the viral genomes. For example, the MCP of *Halorubrum* virus CGΦ46 was encoded on a metagenomic contig also containing genes homologous to the temperate *Halorubrum* virus BJ1. Another virus detected in the metaproteome showed identities to viruses and cellular genes of the HCTV-1 subgroup, as well as the Hlac-Pro1 provirus of *H*. *lacusprofundi,* which, in turn, has sequence similarity to BJ-1 [32,50]. These head-tailed virulent haloarchaeal viruses in Deep Lake likely play a role in nutrient remobilization, and their proposed ability to infect a broad range of hosts likely serves to redistribute DNA within the populations thereby generating genetic diversity in this relatively simplified hypersaline lake system.

Viruses associated with bacterial, algal, and protist hosts are also found in Antarctic sea ice [51]. As sea ice forms, brine channels and pockets enriched in salts and nutrients are created and remain in a liquid state inside the ice. Being semi-enclosed, these support microbial growth and favor high contact rates between viruses and microbial hosts [52]. High viral abundances have been detected in sea ice in various regions in Antarctica, for example, 10^6^–10^8^ viruses mL^−1^ were detected in late autumn and summer in the Ross Sea pack ice [53] that include large viruses with capsid diameters >110 nm in brine and slush ice core samples [51]. Viruses numbering in the range of 0.6–5 × 10^5^ were detected in fast ice samples collected over an annual sea ice cycle from two different depths at two offshore sites in Prydz Bay, East Antarctica [54]. Recently, the first cultivable phage–host systems were isolated from first-year and second-year winter-sea ice from the Weddell Sea [52]. This study identified 59 bacterial strains belonging to nine genera with members of *Paraglaciecola* and *Glaciecola* being the most abundant. Four cold-active tailed bacteriophages of the order *Caudovirales* were isolated on *Paraglaciecola* and *Octadecabacter* strains. These were named for the bacteria of isolation, *Paraglaciecola* Antarctica GD virus 1(PANV1), *Paraglaciecola* Antarctica JLT virus 2 (PANV2), *Octadecabacter* Antarctica BD virus 1 (OANV1), and *Octadecabacter* Antarctica DB virus 2 (OANV2). Transmission electron microscopy (TEM) identified PANV1 as a myovirus and PANV2 as a siphovirus based on tail length and head diameter. Both of these could infect 10 of the 16 *Paraglaciecola* isolates, while two *Paraglaciecola* isolates could be infected by only one of them, PANV1 or PANV2. OANV1, a siphovirus, had a broader host range with the ability to infect two *Octadecabacter* strains (class Alphaproteobacteria) and a *Paraglaciecola* strain (class Gammaproteobacteria). In contrast, OANV2, a podovirus, could only infect one *Octadecabacter* strain.

### 2.2. RNA Viruses in Antarctica

The first discovery of RNA viruses from an Antarctic aquatic environment was reported from Limnopolar Lake [30]. Pyrosequencing was performed on RNA viromes from three sampling seasons; when the lake was ice-covered in the spring of 2006 and when ice-free during the summers of 2007 and 2010. Most of the identified viruses were +ssRNA assigned to the order *Picornavirales* [30]. *Dicistroviridae* were most represented in samples from 2006 to 2010, while the genus *Bacillarnavirus* and *Picornavirales* were found in summer samples of 2007 and 2010 [30]. Four near full-length viral genomes were assembled, named Antarctica picorna-like virus (APLV)1–4 due to their similarity to *Picornavirales* [30]. APLV-1 was identified as a possible arthropod-infecting virus as it clustered with members of the *Dicistroviridae* whose hosts are arthropods. APLV-2, -3, and -4 clustered with *Bacillarnavirus*, *Marnaviridae*, and *Labyrnavirus*, respectively, members of which infect microbial eukaryotes viz. diatoms, algae, and protists, which is consistent with the microbial constituents of Limnopolar lake. APLV-1 was the most abundant virus during the sampling years and displayed little genetic variation, while APLV-2 and APLV-3, seen only in the summer months and also detected in cyanobacterial mats, displayed a quasispecies structure [30].

Similarly, most RNA viruses sampled in surface coastal water viromes along the WAP near Palmer Station were classified as non-enveloped *Picornavirales* [35]. These undergo a seasonal change in abundance, from ~8% in the late-austral spring to 65% in the mid-austral summer. While the summer months (December–March) were dominated by the genus *Bacillarnavirus*, the late spring was dominated by members of the genus *Labyrnavirus* and the family *Dicistroviridae* [35]. Genomic configuration and RNA-dependent RNA polymerase (RdRp)-based phylogenetic placement of five completely assembled genomes indicated that they likely infect diatom species [35], an assessment consistent with seasonal dynamics of measured phytoplankton species in the austral summer months [20,22]. This study is one of the first to uniquely focus on understanding RNA virus diversity in the coastal Antarctic waters, which is still largely unknown [35].

## 3. Viruses in the Arctic

The Arctic undergoes similar seasonal fluctuations in light and freshwater inputs from melting ice as Antarctica [55]. Since temperature and light influence microbial diversity in polar regions, studies have been performed to correlate the viral composition with prokaryotic abundance in order to understand nutrient cycling [55,56]. Viral community composition and distribution in the Arctic has been assessed using molecular methods like Randomly Amplified Polymorphic DNA PCR (RAPD-PCR) [55], and Terminal Restriction Length Polymorphism (T-RFLP) [56] and metagenomics [31,38,55]. In the Canadian Arctic, variation in the viral community with DNA genomes, detected using CRA-22 and OPA-13 primers, is influenced by spatial distance and spatially correlated environmental parameters. In Baffin Bay and the Arctic Archipelago, this variation is uncoupled from the prokaryotic composition, perhaps indicating these viruses infect eukaryotic hosts [56]. Similarly, the coastal region of Kongsfjorden in the Svalbard Archipelago undergoes an increase in prokaryotic and virus abundance during the spring-to-summer transition due to changes in the salinity, density, and temperature of surface waters. However, viral abundance in this region is lower than that seen in lower-latitude waters and Antarctica, due to high particle load, an increase in UV radiation, and the presence of eukaryotic phytoplankton [55]. High viral abundances (7–30× more than prokaryotes) were reported in the Barents Sea [57] even at the onset of the “polar night” at the beginning of winter when there is an increase in heterotrophy [57,58]. High viral abundances were also detected in sea ice in the Amundsen Gulf, approximately two orders of magnitude higher than bacterial concentrations [59]. However, viral diversity is still underexplored in the Arctic, with only a handful of published molecular-based studies thus far, see Figure 1b for sampling sites in the Arctic mentioned in this review.

### 3.1. DNA Viruses in the Arctic

Similar to Antarctica, the freshwater lakes in the Arctic are oligotrophic environments with low predation from metazoans. ssDNA viruses dominated the viromes of six freshwater bodies of the Svalbard Archipelago, Spitsbergen, that were sampled over three years [31]. However, as the viromes were amplified using Phi29 polymerase, ssDNA viruses were likely relatively overrepresented. Of the 9.8% HiSeq Illumina sequencing reads that could be assigned to known taxa, 86% were ssDNA viruses containing a large fraction of *Circoviridae* (38.1%), and 2.8% were dsDNA viruses largely composed of *Caudovirales*-like sequences (1.8%) [31]. While tblastx-derived analysis (*E*-value < 10^−3^) indicated that the most abundant viruses were ssDNA viruses (*Circoviridae*, unclassified ssDNA viruses, *Microviridae*, and *Nanoviridae*), the relative abundances of these individual groups varied based on location and spring/summer seasons. tBlastx and a reference-independent cross-assembly analysis, crAss, were also used to compare the similarity between assembled metagenomic reads of these Arctic lakes with virome reads from published viromes of the Arctic Ocean, Antarctica, Sahara Desert perennial ponds, French temperate lakes, and an aquaculture facility. A clear distinction was found between polar and non-polar viromes, with only a 7.7% similarity between the Arctic and Antarctic samples, and very low similarity between Arctic freshwater viromes and Arctic ocean viromes, clearly delineating freshwater and salt-water communities [31].

The prasinophyte *Micromonas* dominates the picophytoplankton in marine Arctic waters. The polar ecotype has been designated as the species *Micromonas polaris* [60], adapted to grow at temperatures of 0–12 °C and distinct from lower latitude species. *M. polaris* is readily infected by MpoVs, lytic dsDNA prasinoviruses, which are among the most abundant marine phycodnaviruses [61]. *M. polaris* strain TX-01 was isolated in 2014 from Kongsfjorden, an inlet fjord of the Svalbard islands along with four virus strains that infect it: MpoV-44T (isolated in winter 2006), as well as MpoV45T, MpoV-46T, and MpoV47T (isolated spring and summer of 2014 and 2015) from Kongsfjorden and Storfjorden [62]. TEM indicated that all four viruses were morphologically similar with icosahedral capsids ~120 nm in diameter possessing an outer lipid membrane [62]. The four viruses displayed broad host ranges, infecting various Arctic *Micromonas* species in addition to TX-01, even though MpoV45T, MpoV-46T, and MpoV47T had smaller genomes (191 ± 3 kb) than MpoV-44T (205 ± 2 kb), these were distinct from each other and other isolates of Arctic *Micromonas* viruses based on a phylogenetic analysis of DNA polymerase B (DPOB). The infection dynamics of these viruses varied based on differences in temperature from −1 to 7 °C, an ecologically relevant temperature range that is similar to that of Arctic waters transitioning from spring to summer. In general, this caused a decrease in the latent periods and an increase in the burst sizes of these viruses. The dramatic effect of increasing temperature on host growth invokes questions on community composition and the role of viruses in a rapidly warming Arctic Ocean [62].

A tailed dsDNA bacteriophage, 9A, belonging to the *Siphoviridae* family was isolated on *Colwellia psychrerthraea* strain 34H [63]. This cold-active T5-like phage with a genome size of 80–90 kb was isolated from the nepheloid zone in Franklin Bay of the Canadian Arctic. While its native host is 34H (found in the Arctic shelf sediments), it could also infect *C. demingiae* ACAM 459 and could form plaques at a broad temperature range of −6 and 8 °C on ACAM 459, whereas it could only infect 34H grown in a temperature range of −6–4 °C [63]. The study also tested the infectivity of 9A on these two hosts, varying other parameters like salinity, pressure, and starvation, elegantly pointing to the phenotypic plasticity of the phage in differing environmental parameters and hinting at novel molecular mechanisms underlying the interactions between phages and hosts.

A spatial and seasonal survey of the Beaufort Sea and Amundsen Gulf in the Canadian Arctic indicated the composition and dynamics of T4-like myoviruses and phycodnaviruses in these highly heterogeneous Arctic environments [64]. PCR-denaturing gradient gel electrophoresis (DGGE) fingerprinting was performed on environmental samples collected throughout the year (2003–2004) using the following well-established marker genes: DPOB for phycodnaviruses, MCP (*g23*) gene for myophages, along with bacterial (16S) and eukaryotic (18S) SSU rDNA. This indicated patterns in the viral assemblages of the two virus groups in relation to seasonal changes. Fingerprinting of DPOB indicated significantly different phycodnavirus populations according to the season. Phycodnavirus diversity correlated with the 18S rDNA dynamics indicative of changes in the viral population structure and potential host community in response to environmental changes. The g23 banding patterns were highly dynamic, indicating diverse myoviruses infecting prokaryotes differed both seasonally and spatially; but no significant differences in the number of amplicons were detected across the seasons, regions, or depths sampled [64]. While this study provided a qualitative assessment of microbial and viral community structure, it is limited to only two DNA viruses for which primers can be designed and by the resolving power of DGGE, where amplicons of similar size and nucleotide composition migrate together.

ssDNA phages and prophages were identified by pyrosequencing of uncultured viral assemblages derived from 56 samples at 16 different sites in the North-American Arctic Ocean. Assembled sequences from the Arctic and three other marine regions (Sargasso Sea, British Columbia Coast, and Gulf of Mexico) were compared to the nonredundant and environmental SEED databases using tBlastX and tBlastN, and taxonomically assigned using the Phage Proteomic Tree [65]. These analyses suggested marine phages were distinct from phages found in soil and sediments and could be distinguished by their biogeography [38].

Recently, a ssDNA filamentous phage, f327, of the *Inoviridae* family was isolated from a *Pseudoalteromonas* strain, a predominant bacterial genus found in Arctic sea ice [66]. Filamentous phages are known to cycle between a double-stranded plasmid-like replicative form (RF) and an integrated form (IF) in the host cell, but mature virions contain ssDNA [67]. Sequence alignment of genes of this phage revealed ORFs associated with replication, structure, assembly, and transcriptional regulation [66]. Of the 53 strains of *Pseudoalteromonas* isolated from sea ice, 19 contained f327 or f327-like genes indicating both the host and the phage are widespread. Since sea ice brine channels are deficient in nutrients, highly saline and, therefore, limit bacterial growth, the phage is hypothesized to induce motility of the host towards more suitable environments for growth and hence may regulate the host community [66]. Virus-induced mortality is a governing factor for producing nutrients such as amino acids and dissolved DNA, where the latter is readily picked up as a source of phosphorus in nutrient-limited waters and may regulate nutrient availability in the Arctic similar to what is seen in the Baltic Sea [68]. However, the filamentous phage f327 subverts the idea of viruses as simple agents of host mortality, showing they may instead increase host survival.

Recently, pyrosequencing of viral DNA from deep-sea Arctic sediments (5,571 m depth) indicated that ~7% of the annotated reads were taxonomically affiliated with viruses [69]. While dsDNA viruses of the order *Caudovirales* dominated in the virome, a limited number of genotypes associated with ssDNA and RNA viruses were also identified [69]. These Arctic viromes also displayed the highest number of putative functions in comparison with viromes from the Black Sea, North-East Atlantic, and the Mediterranean Sea, indicating diverse viral genotypes that may confer advantages for host–virus interactions [69].

A recent report from an aquatic region next to the Arctic, the Baltic Sea, provides an example of an exhaustive metagenomic and metatranscriptomic analysis of virioplankton and associated microbial taxa in the transition from Arctic to sub-Arctic regions [70]. This region has also been explored for prokaryotic and viral diversity in brine channels formed in sea ice. Cultivation-based methods have recovered *Shewanella* and *Flavobacterium* strains as the most abundant heterotrophic bacteria in the sea ice of the Baltic Sea, where the northern part (Bothnian Bay) may be ice-covered for over six months in severe winters. These cold-active bacteria were reported to be typically infected by tailed siphoviruses and myoviruses with narrow host ranges [71,72]. Six of these isolated phages fall into five new genera based on ICTV criteria for classification [72]. Such cultivation-based studies seem limited in their capacity to assess viral diversity compared to metagenomics, yet these characterized viruses are essential additions to sequence databases, given that more than 60% of metagenomic reads cannot be assigned to known taxonomic groups [16,73].

### 3.2. RNA Viruses in the Arctic

There is limited knowledge of RNA viruses in the Arctic since RNA is often not targeted in environmental sequencing studies; RNA is less stable and thus more difficult to analyze than DNA, and the detection of RNA viruses is hindered by their small genome sizes. ssRNA viruses were reported in freshwater Arctic viromes from six lakes in Spitsbergen. These viruses encoded Rep proteins that most closely resembled those of *Circoviridae-Geminiviridae-Nanoviridae*, with coat proteins similar to the protist pathogen *Scleropthora macrospora* virus A [31]. RNA-Seq methods recently identified both dsRNA and ssRNA viruses in the Baltic Sea Virome [70], indicating the potential for such an approach to be applied to the unexplored RNA virus diversity in the Arctic. This study used RdRp as a reference gene for phylogenetic placement, which showed dsRNA sequences and marine algal viruses had the greatest taxonomic representation. *Retroviridae*, *Picornavirales* (ssRNA), and *Mononegavirales* (−ssRNA) and *Ourmiavirus* (+ssRNA) were also detected [70].

## 4. Conclusions

Both the Arctic and Antarctica are teeming with microbial life spanning the three domains. Given the diversity of mechanisms employed by viruses to exploit their hosts, viruses have been rightfully thought to have “deceptive simplicity”. The studies described in this review indicate that sampling sites in the two poles have viral communities of similar taxonomic composition at higher taxonomic ranks, but are dominated by different viral species. Sequence data provides a more in-depth and thorough assessment of diversity over traditional cultivation-based tools and morphological classification using electron microscopy. As in all metavirome studies, a large number of sequences cannot be taxonomically ascribed—it is common that viral sequences obtained from metagenomes do not match sequences in known databases [17]. This number has been seen to be as high as 91% [38] leading this immense genetic diversity to be referred to as viral dark matter, and hence an enigmatic virosphere [73]. In part, this is an overall consequence of short-read Next-Generation Sequencing—the rate of obtaining genomic sequences has increased, but several factors limit the identification of truly viral sequences and their taxonomic assignment. A key reason is that viruses lack a universal marker gene that can be suitably used to ascribe taxonomy [73]. Additionally, Next-Generation short-read aligners do not allow many mismatches and short reads will only encode partial ORFs. Hence, divergent viruses and viruses with high mutation rates cannot be assigned. Consequently, virus species that may have amino acid similarity to reference genomes are missed [73]. Horizontal gene transfer by viral transduction in extreme cold conditions works to complicate taxonomic assignments [74]. In addition, methodological biases that skew the relative abundances of viral genotypes are acknowledged in understanding virus diversity, for example, the use of the Phi29 polymerase overestimates ssDNA genomes [31].

Are virus reads correctly placed in the correct taxonomic group? Based on some recent reports, one may wonder if taxonomic classification based on similarity searches are accurate as an outcome of computational caveats. First, a recent paper highlights a misunderstanding of the “max_target_seqs” parameter of the NCBI BLAST suite if used to infer the taxonomic origin of a sequence. Even though BLAST will return “N” hits if the value of “max_target_seqs” is set to “N”, these hits may not be the best database matches. BLAST uses “N” to prune the search space, thus it is challenging to correctly estimate the right “N” such that all the related database hits are covered. This is a potential source of error in taxonomic placement, not only of viruses but of any sequence [75]. Second, not all environmental microorganisms and viruses have sequenced relatives in databases. Hence, sequence-similarity-based methods will fail to identify such sequence reads. Moreover, for species with relatives in the database, figuring out which of the database hits are related for taxonomic assignment is a non-trivial task. Recently a two-step BILD (Bayesian Integral Log Odds)-score method has been proposed to identify which of the top BLAST hits are relevant for assigning taxonomy [76]. Perhaps such advances in computational tools will aid in deciphering the large pool of existing metagenomic reads.

RNA viruses remain underexplored in the poles. It has been recognized for about a decade now that methodological constraints usually do not recover RNA viruses, particularly those that lack capsids, extracellular particles, and have small genomes [77,78,79]. Indeed, most polar virome studies discussed in this review have predominantly used methods that recovered DNA viruses. Cultivation-free methods, better isolation techniques, and better sequencing technologies, for example, using HiSeq Illumina that allow fine-grained analyses of marine viromes, continue to be developed [69] and may better uncover the diversity of polar viruses. In particular, dsRNA genomes have been difficult to recover due to limitations of reverse transcriptase in amplifying the terminal regions [70,78].

Archaea remains another exciting and underexplored microbial domain in the polar environment. Although archaea (Crenarcheota and Euryarcheota) have been detected in Arctic sea ice, Arctic seawater, and hypersaline Antarctic lakes [4,80,81], thus far there is little knowledge of viruses infecting archaea in the Arctic. In particular, one wonders at the lack of archaeal RNA viruses. Intriguingly, the discovery of a putative archaeal RNA virus sequence in acidic hot springs of Yellowstone National Park [82] spurs curiosity to further investigate these viruses in the polar archaeal world.

Many regions of both the poles are underexplored in terms of microbial diversity with no known viruses assigned to the prevailing microbial system, mostly due to the challenges of reaching sampling sites and the short time periods permitting access to sampling. For example, Lake Vostok is a sub-glacial lake system covered by 3700 m of ice for the last 15–35 million years. However, recent extraction of ice cores from the lake show that it is not sterile. This oligotrophic system comprises Firmicutes, Actinobacteria, Cyanobacteria, and Proteobacteria as the most abundant bacterial phyla, and the fungi Ascomycota and Basidiomycota as the most abundant eukaryotes [83,84]. The lake system has a complex water chemistry that may have built zones and habitats over millions of years [84]. Such polar systems provide hotbeds for viral discovery since viral-lysis-driven nutrient cycling likely supports the growth and succession of cold-active microbial life [83].

Metagenomics is a powerful tool for exploring microbial and viral diversity, but host–virus relationships will likely be an end goal to understanding biogeochemical and evolutionary processes at the poles. The outstanding question, “which virus infects what host?” stems in part from the inability to cultivate the appropriate hosts. One way around this limitation is highlighted in recent work that uses a combination of metagenomic and metatranscriptomics analyses along with “sequence-based metrics of infection potential and host richness” that will allow evaluation of both taxonomic richness and the recovery of small genomes [70]. The polar aquatic regions promise an exciting avenue for discoveries in habitats that still largely harbor only microbial life, in many instances reminiscent of the primordial microbial world, and may provide exciting opportunities to discover the role of viruses in the evolution of complexity.

## References

[1] Boetius A., Anesio A.M., Deming J.W., Mikucki J.A., Rapp J.Z. (2015). Microbial ecology of the cryosphere: Sea ice and glacial habitats. Nat. Rev. Microbiol..

[2] Rampelotto P.H. (2014). Polar microbiology: Recent advances and future perspectives. Biology.

[3] Laybourn-Parry J. (2009). No place too cold. Science.

[4] Cavicchioli R. (2015). Microbial ecology of antarctic aquatic systems. Nat. Rev. Microbiol..

[5] Anesio A.M., Bellas C.M. (2011). Are low temperature habitats hot spots of microbial evolution driven by viruses?. Trends Microbiol..

[6] Anesio A.M., Laybourn-Parry J. (2012). Glaciers and ice sheets as a biome. Trends Ecol. Evol..

[7] Ghiglione J.-F., Galand P.E., Pommier T., Pedrós-Alió C., Maas E.W., Bakker K., Bertilson S., Kirchman D.L., Lovejoy C., Yager P.L. (2012). Pole-to-pole biogeography of surface and deep marine bacterial communities. Proc. Natl. Acad. Sci. USA.

[8] Herron E.M., Dewey J.F., Pitman I.W.C. (1974). Plate tectonics model for the evolution of the arctic. Geology.

[9] Gaina C., Medvedev S., Torsvik T.H., Koulakov I., Werner S.C. (2014). 4d arctic: A glimpse into the structure and evolution of the arctic in the light of new geophysical maps, plate tectonics and tomographic models. Surv. Geophys..

[10] Kanao M., Suvorov V.D., Toda S., Tsuboi S. (2015). Seismicity, structure and tectonics in the arctic region. Geosci. Front..

[11] Adie R.J. (1962). The geology of antarctica. Antarctic Research: The Matthew Fontaine Maury Memorial Symposium.

[12] Amy J.B., Joseph A.M., Joseph D.P., Phillipe C., Fabien J., Wade H.J. (2005). Microbial diversity in a pacific ocean transect from the arctic to antarctic circles. Aquat. Microbial Ecol..

[13] Suttle C.A. (2007). Marine viruses—Major players in the global ecosystem. Nat. Rev. Microbiol..

[14] Middelboe M., Brussaard C.P.D. (2017). Marine viruses: Key players in marine ecosystems. Viruses.

[15] Bergh Ø., BØrsheim K.Y., Bratbak G., Heldal M. (1989). High abundance of viruses found in aquatic environments. Nature.

[16] Roux S., Hallam S.J., Woyke T., Sullivan M.B. (2015). Viral dark matter and virus–host interactions resolved from publicly available microbial genomes. eLife.

[17] López-Bueno A., Tamames J., Velázquez D., Moya A., Quesada A., Alcamí A. (2009). High diversity of the viral community from an antarctic lake. Science.

[18] Säwström C., Lisle J., Anesio A.M., Priscu J.C., Laybourn-Parry J.J.E. (2008). Bacteriophage in polar inland waters. Extremophiles.

[19] Williams T.J., Long E., Evans F., DeMaere M.Z., Lauro F.M., Raftery M.J., Ducklow H., Grzymski J.J., Murray A.E., Cavicchioli R. (2012). A metaproteomic assessment of winter and summer bacterioplankton from antarctic peninsula coastal surface waters. ISME J..

[20] Schofield O., Saba G., Coleman K., Carvalho F., Couto N., Ducklow H., Finkel Z., Irwin A., Kahl A., Miles T. (2017). Decadal variability in coastal phytoplankton community composition in a changing west antarctic peninsula. Deep Sea Res. Part I: Oceanogr. Res. Pap..

[21] Brum J.R., Hurwitz B.L., Schofield O., Ducklow H.W., Sullivan M.B. (2016). Seasonal time bombs: Dominant temperate viruses affect southern ocean microbial dynamics. ISME J..

[22] Li Z., Cassar N., Huang K., Ducklow H., Schofield O. (2016). Interannual variability in net community production at the western antarctic peninsula region (1997–2014). J. Geophys. Res. Oceans.

[23] Saba G.K., Fraser W.R., Saba V.S., Iannuzzi R.A., Coleman K.E., Doney S.C., Ducklow H.W., Martinson D.G., Miles T.N., Patterson-Fraser D.L. (2014). Winter and spring controls on the summer food web of the coastal west antarctic peninsula. Nat. Commun..

[24] Grzymski J.J., Riesenfeld C.S., Williams T.J., Dussaq A.M., Ducklow H., Erickson M., Cavicchioli R., Murray A.E. (2012). A metagenomic assessment of winter and summer bacterioplankton from antarctica peninsula coastal surface waters. ISME J..

[25] Laybourn-Parry J., Pearce D.A. (2007). The biodiversity and ecology of antarctic lakes: Models for evolution. Philos. Trans. Royal Soc..

[26] Tschitschko B., Williams T.J., Allen M.A., Zhong L., Raftery M.J., Cavicchioli R. (2016). Ecophysiological distinctions of haloarchaea from a hypersaline antarctic lake as determined by metaproteomics. Appl. Environ. Microbiol..

[27] Chenard C., Lauro F.M. (2017). Exploring the viral ecology of high latitude aquatic systems. Microbial Ecology of Extreme Environments.

[28] Rastrojo A., Alcamí A. (2018). Viruses in polar lake and soil ecosystems. Adv. Virus Res..

[29] Aguirre de Cárcer D., López-Bueno A., Alonso-Lobo J.M., Quesada A., Alcamí A. (2016). Metagenomic analysis of lacustrine viral diversity along a latitudinal transect of the antarctic peninsula. FEMS Microbiol. Ecol..

[30] López-Bueno A., Rastrojo A., Peiró R., Arenas M., Alcamí A. (2015). Ecological connectivity shapes quasispecies structure of rna viruses in an antarctic lake. Mol. Ecol..

[31] Aguirre de Cárcer D., López-Bueno A., Pearce D.A., Alcamí A. (2015). Biodiversity and distribution of polar freshwater DNA viruses. Sci. Adv..

[32] Tschitschko B., Williams T.J., Allen M.A., Páez-Espino D., Kyrpides N., Zhong L., Raftery M.J., Cavicchioli R. (2015). Antarctic archaea–virus interactions: Metaproteome-led analysis of invasion, evasion and adaptation. ISME J..

[33] Lauro F.M., DeMaere M.Z., Yau S., Brown M.V., Ng C., Wilkins D., Raftery M.J., Gibson J.A.E., Andrews-Pfannkoch C., Lewis M. (2010). An integrative study of a meromictic lake ecosystem in antarctica. ISME J..

[34] Yau S., Lauro F.M., DeMaere M.Z., Brown M.V., Thomas T., Raftery M.J., Andrews-Pfannkoch C., Lewis M., Hoffman J.M., Gibson J.A. (2011). Virophage control of antarctic algal host–virus dynamics. Proc. Natl. Acad. Sci. USA.

[35] Miranda J.A., Culley A.I., Schvarcz C.R., Steward G.F. (2016). RNA viruses as major contributors to antarctic virioplankton. Environ. Microbiol..

[36] Brum J.R., Ignacio-Espinoza J.C., Roux S., Doulcier G., Acinas S.G., Alberti A., Chaffron S., Cruaud C., de Vargas C., Gasol J.M. (2015). Patterns and ecological drivers of ocean viral communities. Science.

[37] Toro M., Camacho A., Rochera Cordellat C., Rico E., Bañón M., Fernández-Valiente E., Marco E., Justel A., Avendaño M., Ariosa Y. (2007). Limnological characteristics of the freshwater ecosystems of byers peninsula, livingston island, in maritime antarctica. Polar Biol..

[38] Angly F.E., Felts B., Breitbart M., Salamon P., Edwards R.A., Carlson C., Chan A.M., Haynes M., Kelley S., Liu H. (2006). The marine viromes of four oceanic regions. PLoS Biol..

[39] Derelle E., Ferraz C., Escande M.-L., Eychenié S., Cooke R., Piganeau G., Desdevises Y., Bellec L., Moreau H., Grimsley N. (2008). Life-cycle and genome of otv5, a large DNA virus of the pelagic marine unicellular green alga ostreococcus tauri. PLoS ONE.

[40] Ducklow H., Clarke A., Dickhut R., Doney S.C., Geisz H., Huang K., Martinson D.G., Meredith M.P., Moeller H.V., Montes-Hugo M. (2012). The marine system of the western antarctic peninsula. Antarctica Ecosystems: An Extreme Environment in a Changing World.

[41] La Scola B., Desnues C., Pagnier I., Robert C., Barrassi L., Fournous G., Merchat M., Suzan-Monti M., Forterre P., Koonin E. (2008). The virophage as a unique parasite of the giant mimivirus. Nature.

[42] Ng C., DeMaere M.Z., Williams T.J., Lauro F.M., Raftery M., Gibson J.A.E., Andrews-Pfannkoch C., Lewis M., Hoffman J.M., Thomas T. (2010). Metaproteogenomic analysis of a dominant green sulfur bacterium from ace lake, antarctica. ISME J..

[43] Madan N.J., Marshall W.A., Laybourn-Parry J. (2005). Virus and microbial loop dynamics over an annual cycle in three contrasting antarctic lakes. Freshwater Biol..

[44] Laybourn-Parry J., Bell E.M. (2014). Ace lake: Three decades of research on a meromictic, antarctic lake. Polar Biol..

[45] Seshadri R., Kravitz S.A., Smarr L., Gilna P., Frazier M. (2007). Camera: A community resource for metagenomics. PLoS Biol..

[46] Zhou J., Zhang W., Yan S., Xiao J., Zhang Y., Li B., Pan Y., Wang Y. (2013). Diversity of virophages in metagenomic data sets. J. Virol..

[47] Fischer M.G., Suttle C.A. (2011). A virophage at the origin of large DNA transposons. Science.

[48] Yutin N., Shevchenko S., Kapitonov V., Krupovic M., Koonin E.V. (2015). A novel group of diverse polinton-like viruses discovered by metagenome analysis. BMC Biol..

[49] Ferris J.M., Burton H.R.J.H. (1988). The annual cycle of heat content and mechanical stability of hypersaline deep lake, vestfold hills, antarctica. Biology of the Vestfold Hills, Antarctica: Proceedings of the Symposium, Hobart, August 1984.

[50] Krupovič M., Forterre P., Bamford D.H. (2010). Comparative analysis of the mosaic genomes of tailed archaeal viruses and proviruses suggests common themes for virion architecture and assembly with tailed viruses of bacteria. J. Mol. Biol..

[51] Gowing M.M. (2003). Large viruses and infected microeukaryotes in ross sea summer pack ice habitats. J. Mar. Biol..

[52] Luhtanen A.-M., Eronen-Rasimus E., Oksanen H.M., Tison J.-L., Delille B., Dieckmann G.S., Rintala J.-M., Bamford D.H. (2018). The first known virus isolates from antarctic sea ice have complex infection patterns. FEMS Microbiol. Ecol..

[53] Gowing M.M., Riggs B., Garrison D.L., Gibson A.H., Jeffries M. (2002). Large viruses in ross sea late autumn pack ice habitats. Mar. Ecol. Prog. Ser..

[54] Paterson H., Laybourn-Parry J.J.P.B. (2012). Antarctic sea ice viral dynamics over an annual cycle. Polar Biol..

[55] De Corte D., Sintes E., Yokokawa T., Herndl G.J. (2011). Changes in viral and bacterial communities during the ice-melting season in the coastal arctic (kongsfjorden, ny- lesund). Environ. Microbiol..

[56] Winter C., Matthews B., Suttle C.A. (2013). Effects of environmental variation and spatial distance on bacteria, archaea and viruses in sub-polar and arctic waters. ISME J..

[57] Venger M.P., Kopylov A.I., Zabotkina E.A., Makarevich P.R. (2016). The influence of viruses on bacterioplankton of the offshore and coastal parts of the barents sea. Russ. J. Mar. Biol..

[58] Shirokolobova T.I., Zhichkin A.P., Venger M.P., Vodopyanova V.V., Moissev D.V. (2016). Bacteria and viruses of the ice-free aquatic area of teh barents sea at the beginning of polar night. Dokl. Biol. Sci..

[59] Collins R.E., Deming J.W.J.P.B. (2011). Abundant dissolved genetic material in arctic sea ice part ii: Viral dynamics during autumn freeze-up. Polar Biol..

[60] Simon N., Foulon E., Grulois D., Six C., Desdevises Y., Latimier M., Le Gall F., Tragin M., Houdan A., Derelle E. (2017). Revision of the genus micromonas manton et parke (chlorophyta, mamiellophyceae), of the type species m. Pusilla (butcher) manton & parke and of the species m. Commoda van baren, bachy and worden and description of two new species based on the genetic and phenotypic characterization of cultured isolates. Protist.

[61] Hingamp P., Grimsley N., Acinas S.G., Clerissi C., Subirana L., Poulain J., Ferrera I., Sarmento H., Villar E., Lima-Mendez G. (2013). Exploring nucleo-cytoplasmic large DNA viruses in tara oceans microbial metagenomes. ISME J..

[62] Maat D.S., Biggs T., Evans C., van Bleijswijk J.D.L., van der Wel N.N., Dutilh B.E., Brussaard C.P.D. (2017). Characterization and temperature dependence of arctic micromonas polaris viruses. Viruses.

[63] Wells L.E., Deming J. (2006). Characterization of a cold-active bacteriophage on two psychrophilic marine hosts. Aqua. Microb. Ecol..

[64] Payet J., Suttle C.A. (2014). Viral infection of bacteria and phytoplankton in the arctic ocean as viewed through the lens of fingerprint analysis. Aqua. Microb. Ecol..

[65] Rohwer F., Edwards R. (2002). The phage proteomic tree: A genome-based taxonomy for phage. J. Bacteriol..

[66] Yu Z.-C., Chen X.-L., Shen Q.-T., Zhao D.-L., Tang B.-L., Su H.-N., Wu Z.-Y., Qin Q.-L., Xie B.-B., Zhang X.-Y. (2014). Filamentous phages prevalent in pseudoalteromonas spp. Confer properties advantageous to host survival in arctic sea ice. ISME J..

[67] Rakonjac J., Das B., Derda R. (2016). Editorial: Filamentous bacteriophage in bio/nano/technology, bacterial pathogenesis and ecology. Front. Microb..

[68] Riemann L., Holmfeldt K., Titelman J. (2009). Importance of viral lysis and dissolved DNA for bacterioplankton activity in a p-limited estuary, northern baltic sea. J. Microb. Ecol..

[69] Corinaldesi C., Tangherlini M., Dell’Anno A. (2017). From virus isolation to metagenome generation for investigating viral diversity in deep-sea sediments. Sci. Rep..

[70] Zeigler Allen L., McCrow J.P., Ininbergs K., Dupont C.L., Badger J.H., Hoffman J.M., Ekman M., Allen A.E., Bergman B., Venter J.C. (2017). The baltic sea virome: Diversity and transcriptional activity of DNA and rna viruses. mSystems.

[71] Luhtanen A.M., Eronen-Rasimus E., Kaartokallio H., Rintala J.M., Autio R., Roine E. (2014). Isolation and characterization of phage–host systems from the baltic sea ice. Extremophiles.

[72] Senčilo A., Luhtanen A.-M., Saarijärvi M., Bamford D.H., Roine E. (2014). Cold-active bacteriophages from the baltic sea ice have diverse genomes and virus–host interactions. Environ. Microb..

[73] Krishnamurthy S.R., Wang D. (2017). Origins and challenges of viral dark matter. Virus Res..

[74] Simmonds P., Adams M.J., Benkő M., Breitbart M., Brister J.R., Carstens E.B., Davison A.J., Delwart E., Gorbalenya A.E., Harrach B. (2017). Virus taxonomy in the age of metagenomics. Nat. Rev. Microb..

[75] Shah N., Nute M.G., Warnow T., Pop M. (2018). Misunderstood parameter of ncbi blast impacts the correctness of bioinformatics workflows. Bioinformatics.

[76] Shah N., Altschul S.F., Pop M. (2018). Outlier detection in blast hits. Algorithm. Mol. Biol..

[77] Culley A.I., Lang A.S., Suttle C.A. (2006). Metagenomic analysis of coastal RNA virus communities. Science.

[78] Urayama S.-I., Takaki Y., Nishi S., Yoshida-Takashima Y., Deguchi S., Takai K., Nunoura T. (2018). Unveiling the rna virosphere associated with marine microorganisms. Mol. Ecol. Res..

[79] Steward G.F., Culley A.I., Mueller J.A., Wood-Charlson E.M., Belcaid M., Poisson G. (2012). Are we missing half of the viruses in the ocean?. ISME J..

[80] Kalanetra K.M., Bano N., Hollibaugh J.T. (2009). Ammonia-oxidizing archaea in the arctic ocean and antarctic coastal waters. Environ. Microb..

[81] Galand P.E., Casamayor E.O., Kirchman D.L., Potvin M., Lovejoy C. (2009). Unique archaeal assemblages in the arctic ocean unveiled by massively parallel tag sequencing. ISME J..

[82] Wang H., Yu Y., Liu T., Pan Y., Yan S., Wang Y. (2015). Diversity of putative archaeal rna viruses in metagenomic datasets of a yellowstone acidic hot spring. SpringerPlus.

[83] Shtarkman Y.M., Koçer Z.A., Edgar R., Veerapaneni R.S., D’Elia T., Morris P.F., Rogers S.O. (2013). Subglacial lake vostok (antarctica) accretion ice contains a diverse set of sequences from aquatic, marine and sediment-inhabiting bacteria and eukarya. PLoS ONE.

[84] Siegert Martin J., Priscu John C., Alekhina Irina A., Wadham Jemma L., Lyons W.B. (2016). Antarctic subglacial lake exploration: First results and future plans. Philos. Trans. Royal Soc. A.

